# Engineered IL13 variants direct specificity of IL13Rα2-targeted CAR T cell therapy

**DOI:** 10.1073/pnas.2112006119

**Published:** 2022-08-08

**Authors:** Lawrence A. Stern, Sharareh Gholamin, Ignacio Moraga, Xin Yang, Supraja Saravanakumar, Joseph R. Cohen, Renate Starr, Brenda Aguilar, Vanessa Salvary, Jonathan C. Hibbard, Anusha Kalbasi, Jennifer K. Shepphird, James O’Hearn, K. Christopher Garcia, Christine E. Brown

**Affiliations:** ^a^Department of Hematology & Hematopoietic Cell Transplantation, Beckman Research Institute, City of Hope National Medical Center, Duarte, CA 91010;; ^b^Department of Immuno-Oncology, Beckman Research Institute, City of Hope National Medical Center, Duarte, CA 91010;; ^c^Division of Biology and Bioengineering, California Institute of Technology, Pasadena, CA 91125;; ^d^Department of Molecular and Cellular Physiology, Stanford University School of Medicine, Stanford, CA 94305-5345;; ^e^Department of Structural Biology, Stanford University School of Medicine, Stanford, CA 94305-5345;; ^f^Department of Radiation Oncology, Jonsson Comprehensive Cancer Center, David Geffen School of Medicine, University of California, Los Angeles, CA 90024;; ^g^HHMI, Stanford University, Stanford, CA 94305-5345;; ^h^School of Medicine, Stanford University, Stanford, CA 94305-5345

**Keywords:** chimeric antigen receptors, CARs, T cells, IL13Rα2, glioblastoma

## Abstract

IL13Rα2 is an attractive target due to its overexpression in a variety of cancers and rare expression in healthy tissue, motivating expansion of interleukin 13 (IL13)–based chimeric antigen receptor (CAR) T cell therapy from glioblastoma into systemic malignancies. IL13Rα1, the other binding partner of IL13, is ubiquitously expressed in healthy tissue, raising concerns about the therapeutic window of systemic administration. IL13 mutants with diminished binding affinity to IL13Rα1 were previously generated by structure-guided protein engineering. In this study, two such variants, termed C4 and D7, are characterized for their ability to mediate IL13Rα2-specific response as binding domains for CAR T cells. Despite IL13Rα1 and IL13Rα2 sharing similar binding interfaces on IL13, mutations to IL13 that decrease binding affinity for IL13Rα1 did not drastically change binding affinity for IL13Rα2. Micromolar affinity to IL13Rα1 was sufficient to pacify IL13-mutein CAR T cells in the presence of IL13Rα1-overexpressing cells in vitro. Interestingly, effector activity of D7 CAR T cells, but not C4 CAR T cells, was demonstrated when cocultured with IL13Rα1/IL4Rα-coexpressing cancer cells. While low-affinity interactions with IL13Rα1 did not result in observable toxicities in mice, in vivo biodistribution studies demonstrated that C4 and D7 CAR T cells were better able to traffic away from IL13Rα1+ lung tissue than were wild-type (WT) CAR T cells. These results demonstrate the utility of structure-guided engineering of ligand-based binding domains with appropriate selectivity while validating IL13-mutein CARs with improved selectivity for application to systemic IL13Rα2-expressing malignancies.

## Significance

Chimeric antigen receptor (CAR) T cell therapy has demonstrated impressive clinical impact. Imperative to CAR design is the optimization of the antigen binding domain, which imparts CAR specificity, and use of natural ligands circumvents the need for engineering new binding proteins. However, natural molecules may have multiple binding partners, some of which are not therapeutic targets. Here, we study CAR specificity using engineered variants of interleukin 13 (IL13) with different levels of selectivity toward the therapeutic target IL13Rα2 relative to the ubiquitously expressed IL13Rα1. Using stringent models, we demonstrate that antigen selectivity strongly influences both cytotoxicity and CAR T cell trafficking in vivo. These studies highlight structure-guided engineering of binding domains and present therapeutic candidates for IL13Rα2-overexpressing malignancies.

Chimeric antigen receptor (CAR)–engineered T cells have invigorated the field of cancer immunotherapy with their proven ability to treat CD19^+^ malignancies in the clinic (1–4) and continuing progress in solid tumors (5, 6). The synthetic CAR imparts T cells with the ability to recognize antigen independent of peptide presentation by major histocompatibility complexes. This antigen recognition is most often mediated by single-chain variable fragments derived from monoclonal antibodies. As an alternative, naturally occurring ligands or receptors have been used for CAR antigen recognition (7), including interleukin 13 (IL13) (8–10), a proliferation-inducing ligand (APRIL) (11), NKG2D (12), NKp44 (13), and CD27 (14). By leveraging natural binding interactions, these molecules can mediate CAR antigen recognition with minimal additional engineering (8, 14), are fully human in sequence and thus carry potentially lower immunogenicity than other classes of engineered antigen binding domains, and can potentially target multiple cancer biomarkers (11–13). However, the ability to target multiple receptors can also be disadvantageous when binding partners are not implicated in disease.

IL13 is one prominent example of a naturally occurring ligand that has been used for CAR antigen recognition (8–10). IL13 interacts strongly with the high-affinity receptor IL13Rα2 (15), which is a versatile therapeutic target due to its rare expression in normal tissue (16) and overexpression in many human cancers, including glioblastoma (GBM) (17), pancreatic ductal adenocarcinoma (18), melanoma (19), ovarian carcinoma (20), clear cell renal cell carcinoma (21), breast cancer (22), and lung cancer (23). A second IL13 receptor family member, IL13Rα1, interacts with IL13 with lower affinity (15) and is ubiquitously expressed in healthy tissue (16). Additionally, IL4Rα can stabilize the IL13Rα1-IL13 complex (15) to mediate signaling through the JAK/STAT6 pathway (24). This receptor pair is coexpressed in pulmonary and other normal tissues (25). Despite this wide expression of IL13 binding partners in healthy tissue, an IL13 ligand–based CAR has shown safety in humans during clinical trials with locoregional central nervous system delivery in GBM (5, 26), suggesting that toxicity from on-target/off-disease binding is not problematic in this context. However, for the treatment of systemic disease, the wide expression of IL13 binding partners outside of the diseased tissue could act as a sink for IL13-based therapy, resulting in safety concerns and possibly impeding trafficking to the disease site. Previous work in the field has attempted to address this problem by generating CARs derived from IL13 variants containing mutations to direct binding away from IL13Rα1/IL4Rα. Mutations at E12 have yielded improved selectivity for IL13Rα2 over IL13Rα1 (8, 9), albeit with the E12Y mutation still allowing measurable recognition of IL13Rα1 in the context of both recombinant antigen and antigen-expressing cancer cells (9). The addition of both E12K and R109K mutations into an IL13-based CAR also showed attenuated, but not abolished, recognition of IL13Rα1-expressing cancer cells relative to IL13Rα2-expressing cancer cells (10). While these examples are encouraging, additional protein engineering is warranted to develop an IL13Rα2-specific IL13 mutant.

Structure-based protein engineering and directed evolution approaches offer opportunities to modify the affinity and specificity of binding interactions (27, 28). In this approach, structural information is used to identify residues that contribute to binding interactions, combinatorial libraries are developed through designed or random mutation at the identified residues, and high-throughput in vitro methods are employed to screen for the desired function. Previous applications of this method in the context of cytokines have led to the development of a panel of IL13 mutants with a 6-log affinity range for IL13Rα1 to study the interplay of binding affinity and signal transduction (29), engineering of an orthogonal interleukin 2 (IL2) cytokine-receptor complex system that does not act with the native cytokine or receptor (30), and the development of transforming growth factor beta (TGFβ)-based inhibitors (31), among other examples.

Here, we describe the development of IL13-mutein CARs with improved selectivity for IL13Rα2 relative to IL13Rα1 and study their activity in IL13Rα1-expressing, IL13Rα2-expressing, and IL13Rα1/IL4Rα-coexpressing contexts. Prior knowledge of the structures of the IL13 complexes with IL13Rα2 and IL13Rα1/IL4Rα (15) informed the design of an IL13-mutein library that was screened using yeast surface display for diminished binding to IL13Rα1 (29). Characterization of hits yielded two promising candidates, termed C4 and D7, with markedly improved selectivity for IL13Rα2, as shown by affinity characterization. These IL13 muteins were then built into CAR constructs for functional comparison to CARs derived from IL13 wild-type (WT) and IL13 with the E12Y mutation. In vitro and in vivo functional characterization of C4 and D7 IL13-mutein CAR T cells showed decreased activation, degranulation, cytokine release, and cytolytic activity compared to WT and E12Y CAR T cells in the presence of IL13Rα1-expressing cancer cells. Interestingly, C4, but not D7, showed attenuated cytotoxicity relative to WT against IL13Rα1/IL4Rα-coexpressing cancer cells in vitro and in vivo. Conversely, all of the IL13-mutein CAR T cells exhibited similar cytolytic killing of IL13Rα2 targets in vitro and in vivo. Collectively, this work provides insight into the interplay of binding affinity and selectivity in CAR T cell activity and validates IL13-mutein CARs with improved recognition profiles for targeting IL13Rα2-expressing malignancies. Application of these CARs could expand the therapeutic window for systemic administration of IL13Rα2-targeted therapy for a variety of cancers.

## Results

### IL13 C4 and D7 Mutants Show Selective Binding to IL13Rα2.

**Fig. 1.**
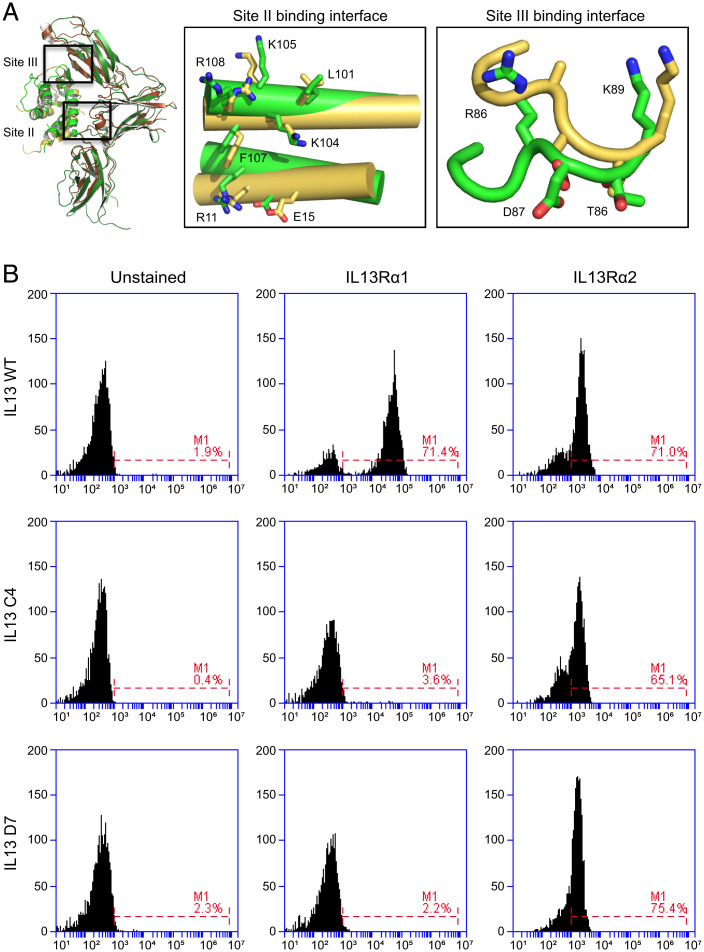
Binding profile of C4 and D7 for IL13 receptors. (*A*) Structural superposition of the IL13/IL13Rα1 and IL13/IL13Rα2 complexes. The docking mode of IL13 with IL13Rα1 is essentially identical to its docking mode with IL13Rα2. *Right Inset:* Boxes show amino acids on IL13 forming the IL13Rα1 and IL13Rα2 site II and site III binding interfaces (yellow: IL13 binding IL13Rα1; green: IL13 binding IL13Rα2). IL13 uses the same amino acids to form the IL13Rα1 and IL13Rα2 site II and site III binding interface. (*B*) IL13 WT binds with high-efficiency IL13Rα1 and IL13Rα2. IL13 C4 and D7 specifically bind IL13Rα2.

Previously, a series of IL13 variants was engineered with a wide range of affinities for IL13Rα1 via structure-guided protein engineering (29). Two such variants, termed C4 and D7, demonstrated markedly diminished affinity for IL13Rα1 relative to IL13 WT. These clones were selected from among many candidates due to their decrease by three and four orders of magnitude in IL13Rα1 binding affinity, respectively (Table 1). As IL13Rα1 and IL13Rα2 share very similar binding interfaces on IL13 (Fig. 1*A*), it was unclear whether the same affinity decrease would be mirrored when complexed with IL13Rα2. To assess receptor binding, WT, C4, and D7 were each displayed on the surface of yeast. Yeast were incubated with either recombinant IL13Rα1 or IL13Rα2, and binding was analyzed by flow cytometry (Fig. 1*B*). As expected, WT bound detectably to both IL13Rα1 and IL13Rα2. By comparison, both C4 and D7 showed no detectable binding to IL13Rα1 at the concentration tested while maintaining the ability to detectably bind to IL13Rα2. Surface plasmon resonance (SPR) studies further confirmed these observations (Table 1 and *SI Appendix*, Fig. 1). WT, C4, and D7 all exhibit subnanomolar affinity for IL13Rα2 (0.001, 0.393, and 0.003 nM, respectively). By contrast, the three variants strongly differ in binding affinity to IL13Rα1, confirming previous reporting (29). These differences result in a several-log range of selectivity for IL13Rα2 relative to IL13Rα1, with WT, C4, and D7 exhibiting 4,400-, 92,000-, and 1,400,000-fold stronger affinity for IL13Rα2 relative to IL13Rα1.

**Table 1.** Binding affinity of IL13 variants.

	IL13Rα1				IL13Rα2				Ratio K_d,Rα1_/K_d,Rα2_
	k_a_ (M^−1^ s^−1^)	k_d_ (s^−1^)	K_d_ (nM)	K_d,mut_/K_d,wt_	k_a_ (M^−1^ s^−1^)	k_d_ (s^−1^)	K_d_ (nM)	K_d,mut_/K_d,wt_	
IL13 WT	5.21 × 10^6^	0.022	4.38	1	5 × 10^7^	8.4 × 10^−5^	0.001	1	4,400
IL13 C4	ND	ND	36,000	8,200	1.94 × 10^6^	7.64 × 10^−4^	0.393	393	92,000
IL13 D7	ND	ND	4,100	940	1.77 × 10^7^	5.37 × 10^−5^	0.003	3	1,400,000

k_a_: association kinetic rate constant; k_d_: dissociation kinetic rate constant; K_d_: equilibrium dissociation constant; K_d,mut_/K_d,wt_: ratio of equilibrium dissociation constants for the specified IL13 variant and wild-type IL13; K_d,Rα1_/K_d,Rα2_: ratio of equilibrium dissociation constants for binding of the variant to IL13Rα1 and IL13Rα2; ND: not determined.

### Generation of C4 and D7 IL13-Variant CAR T Cells.

**Fig. 2.**
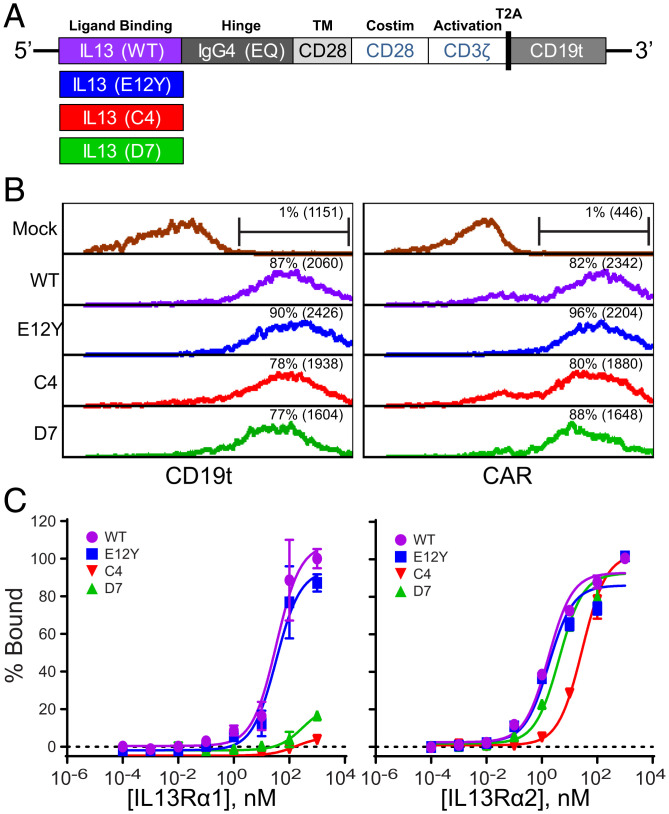
Engineering of IL13-mutein CARs for improved IL13Rα2-selective binding. (*A*) Schematic of IL13-CAR constructs containing WT, E12Y, C4, or D7 binding domains. CARs also include an IgG4(EQ)-Fc linker, a CD28 transmembrane domain (TM) and costimulatory domain (Costim), and a CD3ζ cytoplasmic signaling domain. Constructs also contain CD19t separated from the CAR by a ribosomal T2A skip sequence. (*B*) Histograms showing anti-CD19 for cell transduction (*Left*) and anti-Fc staining for CAR expression (*Right*) for untransduced (mock) and IL13 WT, E12Y, C4, and D7 CARs by flow cytometry. The percentage of positive cells (and mean fluorescence intensity (MFI)) is denoted in each histogram. One representative experiment is shown. (*C*) Dose-response binding affinity curves for the IL13 WT, E12Y, C4, and D7 CAR T cells to human IL13Rα1-Fc (*Left*) or IL13Rα2-Fc (*Right*). Data are presented as mean ± SD of three independent trials.

We designed second-generation CARs incorporating the C4 and D7 variants to determine their impacts on CAR function and IL13Rα2-specific targeting. The CAR constructs contain a targeting domain consisting of either IL13 WT or an IL13 mutein (E12Y, C4, or D7), an optimized immunoglobulin G4 fragment crystallizable (IgG4-Fc) linker mutated at two sites within the CH2 region (L235E and N297Q; EQ) to reduce Fc receptor binding (32), a CD28 transmembrane domain, and the intracellular signaling domains of CD28 in series with CD3ζ (Fig. 2*A*). Our group and others have observed that IL13-mutein–based CARs with CD28-based costimulation, as compared to 4-1BB costimulation, have more sensitive recognition of IL13Rα1 expression (9, 10, 33). Therefore, in order to better differentiate IL13Rα2 specificity, we used CD28 costimulation as part of the CAR backbone. In addition, CAR cassettes have a T2A ribosomal skip sequence (34) followed by a truncated CD19 (CD19t) used as a marker of lentiviral transduction efficiency, cell tracking, and enrichment. Flow cytometry analysis of CD19t for cell transduction and IgG4-Fc for CAR detection confirms comparable expression for the four IL13-CAR variants (Fig. 2*B*). For all in vitro and in vivo studies, IL13-CARs were engineered in naïve/memory T cells (T_n/mem_). T_n/mem_ are enriched CD62L+ T cells that include both the central memory and the stem cell memory populations, along with naïve T cells.

To assess the functional impact of C-terminal fusion of the IL13 variants to IgG4-Fc, we evaluated their binding affinities to recombinant human (rh) IL13Rα1 and IL13Rα2 in dose-response curves (Fig. 2*C*). T cells stably expressing the indicated IL13-based constructs were titrated with recombinant biotinylated human IL13Rα1 or IL13Rα2. The level of receptor bound to IL13 was measured by flow cytometry. Consistent with the affinity characterization of the soluble muteins (Table 1 and *SI Appendix*, Fig. 1), we found that E12Y (equilibrium dissociation constant Kd: 1.8 nM, 95% CI: 1.0 to 3.7 nM) and D7 (Kd: 4.2 nM, 95% CI: 2.8 to 6.3 nM) CAR T cells bind to IL13Rα2 with similar affinity to WT (Kd: 1.7 nM, 95% CI: 1.2 to 2.4 nM), whereas C4 displayed a weaker affinity (Kd: 29 nM, 95% CI: 23 to 36 nM) (Fig. 2 *C*, *Right*). Notably, the affinities of IL13-derived CARs for IL13Rα2 are two to three orders of magnitude weaker than those of the free proteins. By contrast, C4 and D7 CAR T cells exhibited minimal binding to IL13Rα1 on the assayed concentration range, whereas WT and E12Y had nearly identical affinity (34 nM; 95% CI: 21 to 54 nM for both variants) (Fig. 2 *C*, *Left*). Consistent with our findings about binding characteristics with rhIL13Rα1, C4 and D7 CAR T cells exhibited minimal binding to recombinant mouse IL13Rα1, while WT and E12Y CAR T cells bound mouse IL13Rα1 comparably to rhIL13Rα1 (*SI Appendix*, Fig. 6).

### Functional Characterization of C4 and D7 IL13-Variant CAR T Cells in IL13Rα2 Targeting.

**Fig. 3.**
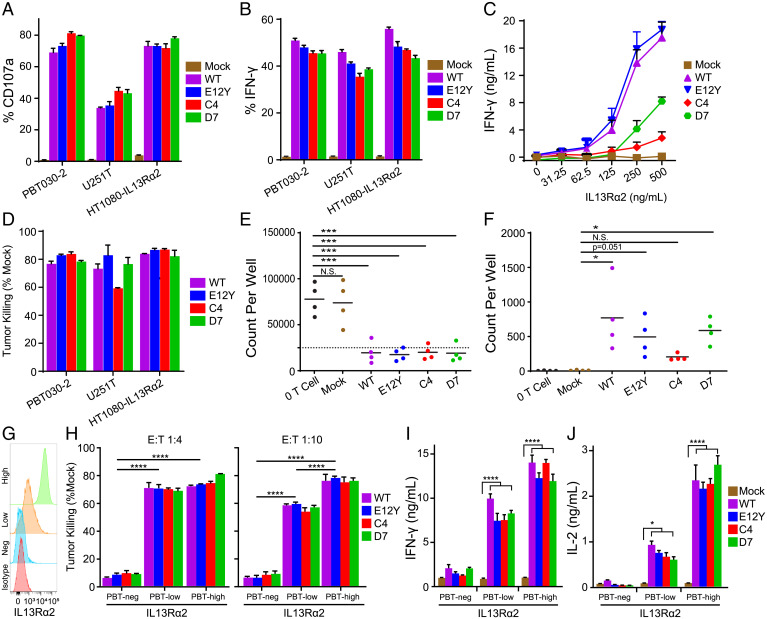
IL13 WT, E12Y, C4, and D7 CARs mediate comparable IL13Rα2-dependent in vitro effector function. IL13Rα2-dependent degranulation (*A*) and IFN-γ production (*B*) for IL13 WT, E12Y, C4, and D7 CAR and untransduced (mock) T cells. T cell lines were cocultured at a 1:1 E:T ratio with IL13Rα2+ GBM lines U251T and PBT030-2 or HT1080 engineered to express IL13Rα2 (HT1080-IL13Rα2); after 5 h, the degranulation marker CD107a and intracellular IFN-γ were measured by flow cytometry, and the percentage of positive cells was graphed (mean ± SD; *n* = 3 wells). (*C*) Secreted IFN-γ production by indicated T cell lines in response to increasing concentrations of immobilized rhIL13Rα2-Fc (mean ± SD; *n* = 3 wells). *P* < 0.0001 for all IL13-CAR T cell variants and mock T cells. (*D*) Tumor killing by IL13-CAR and mock T cell lines cultured at a 1:10 E:T ratio for 2 days. Flow cytometry was used to determine the remaining viable tumor count, and tumor killing was determined as the percentage of normalized to mock (mean ± SD; *n* = 3 wells). (*E*) CAR T cell killing in experiments using four separate donors at a 1:50 E:T ratio against PBT030-2, U251T, and HT1080-IL13Rα2, showing similar levels of tumor killing for WT, E12Y, C4, and D7. Plots represent remaining tumor cells at day 7 (the dotted line represents seeded tumor cells). (*F*) Live CAR T cells in experiments presented following the 7-day coculture presented in *E*. No live CAR T cells were observed in tumor-only and mock CAR T cell experiments; levels of CAR T cell persistence were as follows: WT > D7 > E12Y > C4. *A*–*F* are representative of at least three independent experiments. (*G*) Expression of IL13Rα2 in PBT-neg, PBT-low, and PBT-high cell lines by flow cytometry. (*H*) Tumor killing of PBT-neg, PBT-low, and PBT-high at E:T ratios of 1:4 (*Left*) and 1:10 (*Right*). Noted statistic comparisons for E12Y across groups are representative of trends for all CAR variants. IFN-γ (*I*) and IL2 (*J*) secreted from CAR T cell cocultures with IL13Rα2-engineered cell lines at an E:T ratio of 1:1 were quantified by Luminex assay. Noted statistic comparisons compare each CAR variant individually to mock. All data shown from *A*–*D* are means ± SD and *H*–*J* are means ± SEM (****P < 0.0001, ***P < 0.005, **P < 0.01, *P < 0.05).

We assessed the IL13Rα2-targeting abilities of the IL13 WT and IL13-variant CAR T cells by examining antigen-specific T cell activation. For initial functional studies, we used three IL13Rα2-expressing human cancer cell lines. The patient-derived primary GBM tumor line PBT030-2 and human glioma line U251T endogenously express IL13Rα2 at high levels, consistent with its overexpression in pathological conditions (*SI Appendix*, Fig. 2). The IL13 receptor family-negative human fibrosarcoma cell line HT1080 was engineered to overexpress IL13Rα2 (HT1080-IL13Rα2). IL13Rα2 expression on the cell lines was confirmed by flow cytometry, western blot, and qPCR (*SI Appendix*, Fig. 2). Using CD107a as a marker of degranulation, we evaluated CAR T cell effector function after coculturing the cells with the three IL13Rα2-expressing cell lines at a 1:1 effector-to-target (E:T) ratio. WT, E12Y, C4, and D7 CAR T cells had comparable CD107a expression against all the IL13Rα2-expressing cell lines, measured as the percentage of positive cells, with negligible CD107a expression seen in mock (untransduced) T cells (Fig. 3*A*). As another measure of T cell activity, we evaluated cytokine production, both intracellular interferon-γ (IFN-γ) production by flow cytometry (Fig. 3*B*) and secreted IFN-γ in response to increasing concentrations of immobilized rhIL13Rα2-Fc (Fig. 3*C*). The percentage of cells positive for intracellular IFN-γ at a 1:1 E:T ratio with the IL13Rα2-expressing cell lines was similar across WT, E12Y, C4, and D7 CAR T cells, with mock T cells producing negligible IFN-γ. By contrast, the levels of secreted IFN-γ as a function of IL13Rα2 concentration varied across the CAR T cell types, with WT and E12Y CAR T cells secreting the highest amounts of IFN-γ, followed by D7 and then C4 CAR T cells (Spearman’s Rho = 0.9 with 500 ng/mL IL13Rα2).

To further investigate functional differences between WT and variant CAR T cells, we performed in vitro tumor killing assays. CAR T cells were cocultured with tumor targets PBT030-2, U251T, and HT1080-IL13Rα2 at a 1:10 E:T ratio for 2 days, and viable remaining tumor cells were counted by flow cytometry, with results expressed as the percentage normalized to the tumor cell count after incubation with mock T cells. All of the CAR T cells (WT, E12Y, C4, and D7) killed the tumor cells with similar efficiency (Fig. 3*D*). The functional characteristics of the CAR T cells were consistent across four distinct healthy donors within each experiment. In an extended long-term killing assay, CAR T cells cocultured at a 1:50 E:T ratio with PBT030-2 had similar cytolytic activity regardless of IL13 variant and across four donors (Fig. 3*E*). Importantly, cytotoxicity observed using IL13-mutein CARs was significantly higher than mock and no T cell controls (*P* < 0.005 in all cases). From the same experiments, analysis of live CAR T cells after 7 days of coculture showed persistence/proliferation in WT and D7 CAR T cells compared with the mock control, in which no live T cells were observed (Fig. 3*F*). E12Y CAR T cells demonstrated persistence/proliferation at the boundary of statistical significance as compared to the mock control (*P* = 0.0505). Of note, C4 displayed lower levels of CAR T cell persistence/expansion that were not statistically significant when compared to the mock control (*P* = 0.70), suggesting reduced potential for antigen-dependent proliferation of this variant. In addition to direct functional outputs, information from these assays using multiple IL13 variants informs experimental sensitivities to binding affinity. In this case, cytokine secretion and T cell proliferation in response to prolonged challenge demonstrate sensitivity to small differences (i.e., one order of magnitude) in antigen binding affinity, while degranulation, intracellular cytokine production, and cytotoxicity show little to no difference based on antigen binding affinity. These observations can help inform which in vitro assays are more predictive of CAR potency.

To expand on these observations, we performed additional in vitro tumor killing and cytokine release assays using a panel of patient-derived brain tumor cell lines engineered to express different levels of IL13Rα2. The parental PBT138 cell line does not measurably express IL13Rα2 (PBT138 negative [PBT-neg]; Fig. 3*G*). Engineered variants of PBT138 were then generated to express low (PBT-low) and high (PBT-high) levels of IL13Rα2, and expression was confirmed by flow cytometry. Coculture experiments using a representative T cell donor at an E:T ratio of 1:4 resulted in all CAR T cell variants killing PBT-low and PBT-high with similar efficiency and significantly more than PBT-neg (*P* < 0.0001 in all cases; Fig. 3 *H*, *Left*). Using a more stringent E:T ratio of 1:10 showed uniform differences in cytotoxic activity for all CAR T cell variants across antigen expression levels, with all variants killing higher percentages of tumor cells with higher IL13Rα2 expression levels (*P* < 0.0001 for all comparisons; Fig. 3*H*). Secretion of the cytokines IFN-γ and IL2 from cocultures with a 1:1 E:T ratio demonstrated specificity for CAR function relative to mock control for all cell lines tested by Luminex assay (Fig. 3 *I* and *J*). These results are corroborated by additional coculture experiments using other solid tumor lines, including OVCAR-8 human ovarian carcinoma and M202 human melanoma that endogenously express IL13Rα2 and IL13Rα2-negative PC3 prostate cancer. Cytotoxicity and cytokine secretion mediated by CAR T cell variants mirror the results shown with engineered cell lines, further establishing the ability of these CAR T cell variants to react to a wide array of cancer types and IL13Rα2 expression levels (*SI Appendix*, Fig. 7). Patient-derived IL13Rα2+ xenograft mouse models demonstrate improved survival after treatment with IL13-mutein CAR T cells.

**Fig. 4.**
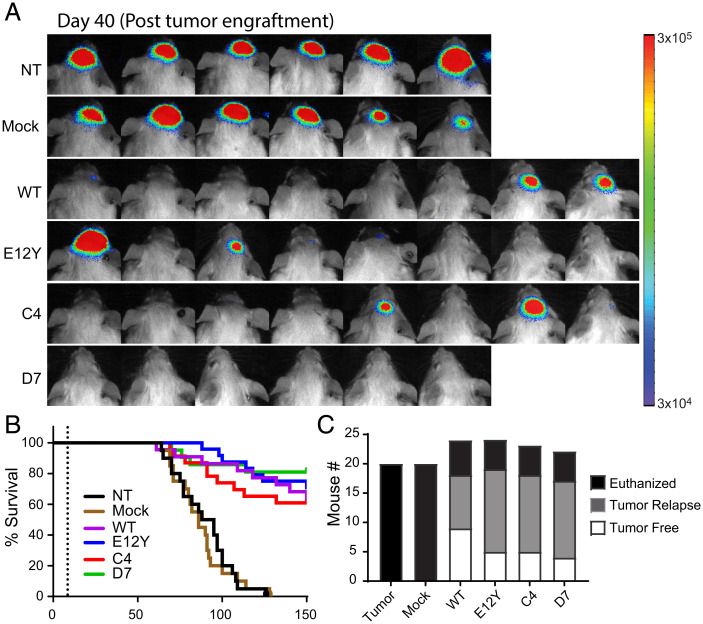
IL13 E12Y, C4, and D7 mutein CARs exhibit similar levels of therapeutic efficacy in vivo against human glioma xenografts. In vivo efficacy is similar across WT and mutein CARs against human glioma xenografts. (*A*) NSG mice stereotactically implanted into the right forebrain with 0.1 × 10^5^ enhanced green fluorescent protein (EGFP)-ffLuc^+^ PBT030-2 tumor spheres. On day 8, mice received no treatment (NT), 0.36 × 10^6^ injection of mock T_n/mem_ (no CAR) T cells, or 0.3 × 10^6^ injection of the following CAR-positive T_n/mem_ cells: IL13 WT, E12Y, C4, or D7 (*n* = 6–8; 2 of the mice in group D7 died early during treatment). Representative flux images are shown on day 150 postengraftment. (*B*) Kaplan-Meier survival curves (summary data for three experiments) demonstrate comparable survival at day 150 (the dotted line represents 9 ± 1 days) for mice treated with the IL13-CAR variants, with IL13 WT, E12Y, C4, and D7 CAR T cells all improving survival as compared to mock T cells (*P* ≤ 0.0001, Mantle-Cox log rank test; *n* = 14–18). (*C*) Bar graph summarizing data from three separate experiments, indicating the number of tumor-free, tumor-relapsed, and euthanized untreated mice and mice treated with the IL13-CAR variants, with IL13 WT, E12Y, C4 and, D7 CAR T cells, at day 150 after tumor implantation.

We evaluated the in vivo antitumor efficacy of the CAR T cells in our previously established xenograft brain tumor model with IL13Rα2-expressing PBT030-2 cells engineered to express the firefly luciferase (ffLuc) reporter gene (35). In three independent experiments, tumor-bearing nonobese diabetic/severe combined immunodeficiency (NOD/Scid) IL2RγCnull (NSG) mice (1 × 10^6^ tumor cells injected intracranially; 9 ± 1 days of engraftment) that received intratumoral (IT) injection of 0.36 × 10^6^ mock (untransduced) T cells exhibited tumor growth and survival similar to nontreated controls, whereas treatment with WT, E12Y, C4, and D7 CAR T cells efficiently reduced tumor burden (Fig. 4*A*). Kaplan-Meier survival analysis demonstrated improved survival for mice treated with IL13 WT and IL13-variant CAR T cells (Fig. 4*B* and *SI Appendix*, Fig. 3), and by day 150, the mice treated with WT and variant CAR T cells had similar numbers of tumor-free and tumor-relapsed mice (Fig. 4*C*). Taken together, these results demonstrate that C4 and D7 CAR T cells exhibit similar in vivo antitumor activity for IL13Rα2-expressing tumors compared with established WT and E12Y CAR T cell treatment.

To interrogate the in vivo persistence of CAR T cell variants, NSG mice were engrafted subcutaneously (s.c.) with IL13Rα2-expressing M202 melanoma cells followed by IT treatment with CAR T cell variants that coexpress ffLuc. Luminescence imaging throughout the 21-day duration of this experiment demonstrates the persistence of all CAR T cell variants at the tumor site (*SI Appendix*, Fig. 4 *A* and *B*). Flow cytometry analysis of T cell infiltrates in harvested tumors reveals comparable levels of all CAR T cell variants at the experimental end point, further demonstrating comparable persistence (*SI Appendix*, Fig. 4*D*).

### IL13-Mutein CAR T Cells Display Diminished Effector Activity against IL13Rα1-Expressing Tumors.

**Fig. 5.**
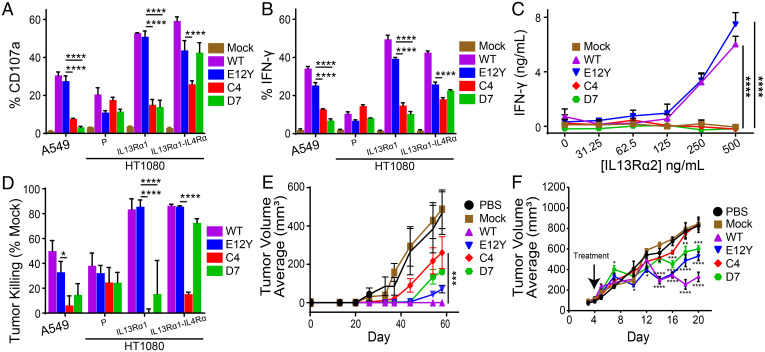
C4 and D7 IL13-mutein CAR T cells showed reduced recognition of IL13Rα1. IL13Rα1-depedendent degranulation (*A*) and IFN-γ production (*B*) for IL13 WT, E12Y, C4, and D7 CAR T cells cocultured with IL13Rα1+ A549, HT1080 parental, or HT1080 engineered to express either IL13Rα1 or both IL13Rα1 and IL4Rα as described in Fig. 3. (*C*) Secreted IFN-γ production by indicated T cell lines in response to increasing concentrations of immobilized rhIL13Rα1-Fc (mean ± SD; *n* = 3 wells). (*D*) IL13Rα1-dependent cell killing of the indicated CAR and target lines at a 1:10 E:T ratio for 2 days. *A*–*D* are representative of at least three independent experiments. (*E*) Winn assay evaluating A549 (0.1 × 10^6^) tumor engraftment after coculture with CAR and mock T cell lines (E:T ratio of 10:1; 2 h preincubation and engraftment on day 0; *n* = 4). (*F*) NSG mice xenotransplanted with 0.5 × 10^6^ HT1080 IL13Rα1/IL4Rα in a 1:1 ratio of media to Matrigel, followed by treatment with no T cells, 5 × 10^6^ mock T cells, or the various mutein CAR T cells on day 4. All data shown on *A*–*F* are means ± SEM (****P < 0.0001, ***P < 0.005, **P < 0.01, *P < 0.05).

Several human cancer cell lines with varying expression of IL13Rα1 were used to evaluate relative effector activity of IL13 WT and IL13-variant CAR T cells. Human lung adenocarcinoma cell line A549 endogenously expresses moderate levels of IL13Rα1 with no detectable IL13Rα2 or IL4Rα. Human fibrosarcoma cell line HT1080, which does not express IL13Rα1, IL13Rα2, or IL4Rα, was engineered to overexpress either IL13Rα1 (denoted HT1080-IL13Rα1) or both IL13Rα1 and IL4Rα (denoted HT1080-IL13Rα1-IL4Rα) (*SI Appendix*, Fig. 2). In coculture of CAR T cells with IL13Rα1-expressing tumor cells (A549 and HT1080-IL13Rα1) at a 1:1 E:T ratio, WT and E12Y induced significantly more surface expression of CD107a (Fig. 5*A*) and intracellular expression of IFN-γ (Fig. 5*B*) than C4 and D7 (*P* < 0.05 for all comparisons). In contrast, when CAR T cells were cocultured with HT1080-IL13Rα1-IL4Rα, E12Y and D7 showed similar expression of surface CD107a and intracellular IFN-γ, whereas C4 displayed an attenuated response relative to E12Y in both assays (*P* < 0.05 for both). Notably, against HT1080-IL13Rα1-IL4Rα cells, all mutein CAR T cells (E12Y, C4, and D7) had a significantly lower frequency of IFN-γ–producing cells compared with WT CAR T cells. Evaluation of secreted IFN-γ after culture with plate-bound IL13Rα1 revealed distinct differences in T cell activity among the CAR variants. WT and E12Y both secreted significantly higher IFN-γ levels compared with C4 and D7, which had nearly undetectable IFN-γ (Fig. 5*C*). The results of long-term killing assays against IL13Rα1-expressing cancer cell lines mirrored those of the degranulation and cytokine production assays: C4 and D7 CAR T cells showed diminished killing relative to WT and E12Y (Fig. 5*D*). Against HT1080-IL13Rα1-IL4Rα, D7 killed comparably to E12Y and WT, whereas C4 showed significantly less tumor killing than E12Y (*P* < 0.0001).

To interrogate the selectivity of the C4 and D7 mutein CAR T cells in vivo, we investigated the antitumor activity of the CAR T cells in xenograft models using IL13Rα1-expressing tumor cells. In order to detect small differences in CAR T cell activity with greater sensitivity, we used the Winn assay to directly evaluate effector activity by incubating tumor and T cells together prior to injection (36, 37). We cocultured 1 × 10^6^ WT, E12Y, C4, and D7 CAR T cells with 0.1 × 10^6^ A549 cells for 2 h, followed by engraftment of the cocultured cells into NSG mice (Fig. 5*E*). Following tumor growth kinetics over 60 days, the WT CAR T cells ablated engraftment of IL13Rα1-expressing tumors. All CAR T cell variants displayed some delay in engraftment relative to phosphate buffered saline (PBS) and mock T cell–treated tumors. At later time points, C4 CAR T cells displayed significantly less growth inhibition than WT and E12Y (*P* < 0.05 for all comparisons on days 54 and 58).

To evaluate the CAR T cell variants against tumors expressing the high-affinity pair IL13Rα1/IL4Rα, NSG mice were xenotransplanted s.c. with 0.5 × 10^6^ HT1080-IL13Rα1-IL4Rα with 4-day engraftment. The mice were treated with mock, WT, E12Y, C4, or D7 CAR T cells or PBS by IT injection (Fig. 5*F*). Again, the C4 CAR T cells had the least amount of antitumor activity, with tumor growth comparable to PBS-treated and mock T cell–treated mice (Fig. 5*F*). Similar to the in vitro killing assays, E12Y and D7 CAR T cell treatment showed comparable decreased antitumor activity relative to WT CAR T cells (*P* < 0.05 for all comparisons for day 16 and thereafter).

### IL13-Variant CAR T Cell Trafficking Is Affected by Endogenous IL13Rα1 Expression.

**Fig. 6.**
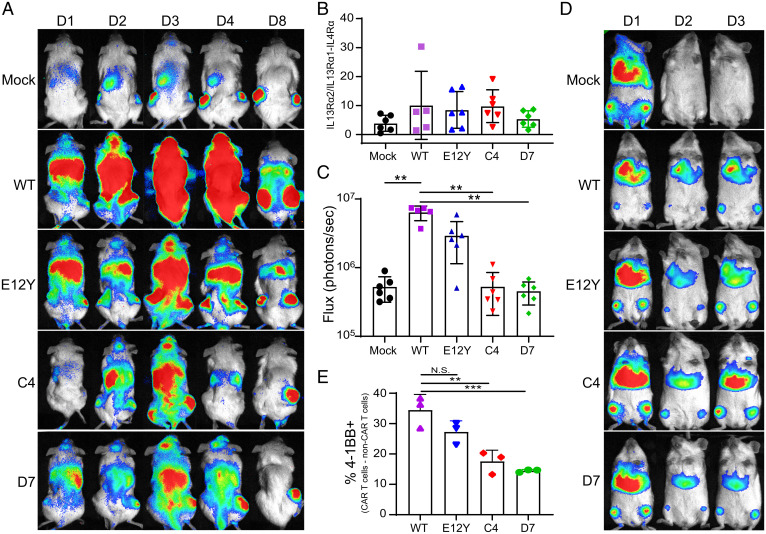
In vivo biodistribution of IL13-variant CAR T cells. (*A*) Mice were xenotransplanted with HT1080-IL13Rα2 tumors in the right flank and HT1080-IL13Rα1-IL4Rα tumors in the left flank prior to i.v. injection of ffLuc-expressing mock or CAR T cells. Luminescence imaging was carried out to track T cell biodistribution. One representative mouse from each group is shown (D1, day 1). (*B*) Ratio of luminescence in the HT1080-IL13Rα2 tumor (right flank) to that of the HT1080-IL13Rα1-IL4Rα tumor (left flank) on day 8 was determined by ROI analysis. Bars show mean ± SD of five to six mice, with data from each individual mouse shown by symbols superimposed on the bars. (*C*) Luminescence of the lungs on day 8 was determined by ROI analysis. Bars show mean ± SD of five to six mice, with data from each individual mouse shown by symbols superimposed on the bars. (*D*) Tumor-free mice were injected with ffLuc-expressing mock or CAR T cells. Luminescence imaging was carried out to track T cell biodistribution. One representative mouse from each group is shown (D1; day 1). (*E*) The frequency of 4-1BB+ CAR T cells (shown as CD19+ T cells minus CD19− T cells) in processed lung tissue was assessed by flow cytometry. Error bars show mean ± SD of three mice, with data from each individual mouse shown by symbols superimposed on the bars (***P < 0.005, **P < 0.01).

To investigate the influence of IL13Rα1 expression in healthy tissue on the trafficking of IL13-variant CAR T cells, we conducted in vivo biodistribution studies in a bilateral tumor model. Using this model for trafficking studies provides an appropriate healthy tissue background. Transcriptome analysis of C57BL/6 mouse tissues shows expression of IL4Rα and IL13Rα1 in several tissues, including liver and lungs, as well as restricted, low-level expression of IL13Rα2 in few tissues (*SI Appendix*, Fig. 5*A*) (38). In NSG mice, qPCR analysis corroborated expression of IL4Rα and IL13Rα1 in liver and lung tissues, as well as minimal expression of IL13Rα2 in liver and lungs (*SI Appendix*, Fig. 5*B*). Human IL13 is known to bind murine IL13Rα1 and IL13Rα2 strongly (9, 33). We verified that WT and variant CAR T cells demonstrated similar ability to bind murine and human IL13Rα1, further validating this model as having appropriate background receptor expression (*SI Appendix*, Fig. 6). NSG mice were engrafted s.c. with HT1080-IL13Rα1-IL4Rα tumors in the left flank and HT1080-IL13Rα2 tumors in the right flank prior to intravenous (i.v.) injection of mock or CAR T cells that express ffLuc. T cell trafficking was assessed by luminescence imaging on days 1, 2, 3, 4, and 8 post–CAR T cell injection (Fig. 6*A*). On day 1 postinjection, CAR T cells appeared to be heavily localized in the lungs of the mice, consistent with other recent reports of CAR T cell biodistribution after i.v. injection (39, 40). Over time, the CAR T cells localized to both flank tumors. Region of interest (ROI) analysis of luminescence in the flank tumors on day 8 revealed all groups of CAR and mock T cells preferentially localized to the HT1080-IL13Rα2 tumor, with no significant change in this preference regardless of CAR variant (Fig. 6*B*). ROI analysis of the lungs on day 8 demonstrated that WT CAR T cells remained in the lungs significantly more than mock, C4, and D7 CAR T cells (Fig. 6*C*; *P* = 0.0001 for all comparisons noted). To further investigate this phenomenon, we performed a similar biodistribution experiment with tumor-free NSG mice. T cell biodistribution was tracked for 3 days by daily luminescence imaging, revealing CAR T cells persisting in the lungs and bone marrow through the experimental end point (Fig. 6*D*). Flow cytometry analysis of excised lung tissue reveals the presence of activated CAR T cells by 4-1BB positivity, with WT and E12Y demonstrating a higher frequency of activated CAR T cells than C4 and D7 (Fig. 6*E*, *P* < 0.05 for all noted comparisons). Importantly, despite this presence of activated T cells in the lungs, no mice were observed to have difficulty breathing through the duration of the experiment and excised lung tissue at the terminal end point did not visually show damage.

## Discussion

CARs using IL13 mutants for targeting IL13Rα2 have previously shown efficacy in both preclinical (8–10) and clinical (5) glioma. Importantly, T cells harboring these CARs have demonstrated safety in their respective clinical settings via intracranial delivery into the resected tumor volume and infusion into the ventricular system (5, 26). In addition to glioma, IL13Rα2 is expressed in a variety of malignancies that could be treated by systemic administration of IL13-CAR T cell therapy. However, IL13Rα1, the other binding partner of IL13, is ubiquitously expressed in healthy tissue (16) and can be bound with low nanomolar affinity (K_d_ = 4 nM). This ubiquitous expression could act as a hindrance to CAR T cell trafficking to malignant tissue, as well as potentially contribute to early T cell exhaustion by inducing many CAR-mediated activation events. In this work, we aimed to increase the therapeutic window of IL13-CAR T cell therapy by investigating IL13 mutants with heavily diminished binding to IL13Rα1 while retaining the clinically effective low nanomolar affinity for IL13Rα2. Our results showed that binding affinity must be drastically diminished to overcome recognition in CAR T cell–cancer cell interactions, which is likely primarily avidity driven.

Understanding the binding interface of IL13 and IL13Rα1 (15) informed the design of a softly mutated combinatorial library from which IL13 mutants C4 and D7, which have markedly diminished affinity for IL13Rα1, were previously isolated (29). Structural studies revealed that IL13Rα1 and IL13Rα2 share a similar binding interface on IL13 (Fig. 1*A*) (15). Interestingly, despite the shared interface, C4 and D7 retained subnanomolar binding affinity for IL13Rα2 comparable to WT (Table 1). Strikingly, when IL13 variants were fused to the IgG4 hinge and Fc at their C termini, their binding affinities for IL13Rα2 were 2 to 3 logs weaker than those of the free proteins (Fig. 2*C*). These differences in affinity could be attributed to restrictions of achievable orientations by the IL13 fusions. Although the IgG4 hinge is flexible, it is only 12 residues long, potentially restricting the orientational freedom that IL13 needs to bind its receptors. Alternatively, fusing additional domains to the C terminus of IL13 alone could affect binding affinity. Other natural molecules, including interleukin 15 (IL15) (41), soluble tumor necrosis factor alpha receptor I (TNFαRI), and soluble TNFαRII (42), have displayed attenuated or abolished biological activity as a result of C-terminal fusion to additional domains. However, interrogating the cause of these differences in affinity is beyond the scope of this study.

Despite differences in affinity for IL13Rα2, all of the IL13-variant CARs demonstrated effector activity when exposed to IL13Rα2-expressing cancer cells and recombinant IL13Rα2 antigen (Fig. 3). When cultured with different amounts of plate-bound recombinant IL13Rα2, the CAR T cells produced IFN-γ in rank order by affinity, with C4 yielding the lowest cytokine production (Fig. 3*C*). Further, C4 CAR T cells displayed significantly lower killing of U251T, a lower expresser of IL13Rα2, than the other three CARs tested (Fig. 3*D*). Experiments with GBM cell lines engineered to express either low or high levels of IL13Ra2 (Fig. 3*H*), along with additional solid tumor lines exhibiting a range of endogenous (*SI Appendix*, Fig. 7 *B* and *C*) IL13Rα2 expression levels, extended these observations, demonstrating that C4 and D7 show comparable in vitro effector function against tumor lines with a range of IL13Ra2 expression levels. In extended long-term killing assays, although they overcame tumor challenge, C4 CAR T cells failed to proliferate/persist significantly better than mock transduced T cells (Fig. 3*F*). Previous experience with affinity optimization of CARs has shown that decreasing affinity can yield decreased effector function in an antigen density-dependent manner (43–45). This phenomenon could explain the differences in effector function observed with C4, which has one order of magnitude weaker affinity than the other three variants tested, but other studies do not show such striking impacts at this level of affinity. Importantly, these in vitro differences were not observed in vivo, with all CAR variants exhibiting similar therapy in an IL13Rα2-expressing human glioma xenograft model (Fig. 4). Additionally, all CAR T cell variants exhibited similar persistence when infused intratumorally in human melanoma–bearing mice (*SI Appendix*, Fig. 4).

Decreasing the binding affinity for IL13Rα1 to the micromolar range had the desired effect in in vitro tumor treatment assays, with both C4 and D7 CAR T cells showing diminished responses relative to WT and E12Y CAR T cells when exposed to IL13Rα1-expressing cancer cells and plate-bound IL13Rα1 (Fig. 5). Similar decreased cellular responses due to micromolar-affinity CAR binding domains have been observed previously in a HER2-expressing model system (45). However, in Winn assays, C4 displayed the least inhibition to IL13Rα1-expressing A549 tumor xenografts. This assay, with a 10:1 E:T ratio, constitutes a very high bar for assessing specificity, as the cocultures are very crowded with CAR T cells, increasing the likelihood of even very weak CAR-antigen interactions. Interestingly, coexpression of IL13Rα1 or IL4Rα in cancer cells caused both E12Y and D7 CAR T cells, but not C4 CAR T cells, to respond comparably to WT CAR T cells in vitro. Although the E12Y mutation eliminates one hotspot residue for binding IL4Rα (34), previous studies have shown that E12Y CAR T cells can still recognize IL13Rα1-expressing cells (9). Neither clones C4 nor D7 have mutations that would diminish IL4Rα binding, so the lack of response by C4 is striking. These results suggest, potentially, that with the weak binding affinity of D7 for IL13Rα1, D7/IL13Rα1 can still form a complex long enough to recruit high-affinity binding with IL4Rα, which then mediates CAR activation. This is not the case for C4, with a 10-fold weaker affinity for IL13Rα1 likely causing the C4/IL13Rα1 complex to be too short lived to recruit IL4Rα. This result is further validated through a s.c. HT1080 model that coexpresses IL13Rα1 and IL4Rα. After IT injection, C4 CAR T cells have no effect on tumor growth relative to mock transduced T cells and PBS alone, while D7, E12Y, and WT CAR T cells mediated significant tumor growth inhibition. It should be noted, however, that this model is designed for high sensitivity with strong overexpression of IL13Rα1 and IL4Rα. The observed trends may be weakened with endogenous expression levels.

In vivo biodistribution studies further demonstrated the importance of tuning CAR binding affinity for endogenously expressed target molecules. In the bilateral tumor model, mock and CAR T cells localized to both tumors, regardless of relative selectivity of the CAR binding domain (Fig. 6*A*). This can potentially be attributed to the chemoattractant nature of the tumors drawing the T cells to both locations in an antigen-independent manner. However, all T cells studied preferentially localized to the HT1080-IL13Rα2 tumors (Fig. 6*B*). It has been shown in other disease contexts that expression of IL13Rα2 contributes to a more aggressive tumor phenotype (22), which could potentially explain this preferential attraction of T cells. The highly retained flux in the lungs of mice injected with WT or E12Y CAR T cells (Fig. 6*C*) suggests that the low-level expression of IL13Rα1 in lung tissue (*SI Appendix*, Fig. 5) is sufficient to retain CARs with high-affinity interactions. However, it should be noted that regardless of T cell retention in the lungs, strong luminescence signals were observed in both flank tumors, suggesting that this phenomenon potentially would not inhibit treatment. Despite potential activation of a subset of the lung-resident T cells demonstrated by 4-1BB expression (Fig. 6*E*), no mice were observed with breathing difficulties and excised lung tissue did not show notable damage, suggesting that lung-resident T cells were not harmful to the healthy tissue. The lower frequency of 4-1BB–expressing C4 and D7 CAR T cells recovered from lungs suggests these muteins with decreased IL13Rα1 affinity guard against premature activation in the presence of endogenous expression relative to WT CAR T cells, which may be advantageous for the therapeutic potential of systemic administration.

## Conclusions

In conclusion, IL13-mutein CAR T cells show improved selectivity for IL13Rα2-expressing cancer cells relative to IL13Rα1-expressing tumors. Previous engineering of IL13 to diminish binding affinity to IL13Rα1 did not drastically affect binding affinity to IL13Rα2, despite the two receptors sharing a similar interface on IL13. Binding affinities in the micromolar range were sufficient to diminish CAR T cell response to IL13Rα1-expressing cells relative to WT CAR T cells. However, the low micromolar affinity was not sufficient to decrease D7 CAR T cell response to IL13Rα1/IL4Rα-overexpressing cancer cells. In vivo biodistribution studies demonstrated the importance of low affinity for receptors expressed at endogenous levels in ensuring CAR T cells traffic away from healthy tissue. Taken together, these results establish the importance of structure-guided protein engineering to designing CAR binding domains with exquisite selectivity and characterize IL13-mutein CAR T cells with enhanced selectivity for IL13Rα2 for potential expansion of IL13-based immunotherapy to malignancies outside of GBM.

## Methods

### Tumor Lines.

PBT030-2 is a patient-derived primary GBM tumor sphere line which was heterotopically passaged twice in NSG mice (35). Established human tumor lines A549 (lung carcinoma, RRID: CVCL_0023), and HT1080 (fibrosarcoma, RRID: CVCL_0317) were obtained from the American Tissue Culture Collection (ATCC) and maintained in Dulbecco's Modified Eagle Medium (DMEM) (Gibco, Thermo Fisher Scientific) supplemented with 10% fetal bovine serum (FBS) (HyClone, GE Healthcare), 2 mM L-glutamine (CAS 56-85-9), and 25 mM HEPES. HT1080 was modified lentivirally to express IL13Rα1, both IL13Rα1 and IL4Rα, or IL13Rα2. The U251T glioma line was a gift from Dr. Waldemar Debinski and grown as previously described (46). Cell line TF-1 (erythroleukemia, RRID: CVCL_0559) was grown in Roswell Park Memorial Institute (RPMI) containing 10% FBS, penicillin-streptomycin, 2 mM L-glutamine, and granulocyte-macrophage colony-stimulating factor (GM-CSF) to promote proliferation and survival. PBT-neg, PBT-low, and PBT-high are variants of a patient-derived GBM tumor engineered to have negative, low, and high expression of IL13Rα2, respectively. M202 (a gift from the Dr. Antoni Ribas laboratory at University of California, Los Angeles) (47) and OVCAR-8 and PC3 (gifts from the Dr. Saul Priceman laboratory at the City of Hope National Medical Center [COH]) were cultured in RPMI containing 10% FBS. All cell lines were maintained at 37 °C with 5% CO_2_.

### Flow Cytometry.

CAR expression was assessed using biotinylated anti-Fc (Jackson ImmunoResearch, 1:100, RRID: AB_2337663) antibody followed by streptavidin-phycoerythrin (PE) (BD Biosciences, 1:20, RRID: AB_10053328) and by staining for the CD19t extracellular sequence with CD19-PE-Cy7 (BD Biosciences, clone SJ25C1, 1:100, RRID: AB_396893). Target lines were characterized by staining with IL13Rα2-PE (BioLegend, clone SHM38, 1:100, RRID: AB_11218806), IL13Rα1 (BioLegend, clone SS12B, 1:100, RRID: AB_2562552), and IL-4Rα-PE (BD Pharmingen, clone hIL4R-M57, 1:20, RRID: AB_394355). In other assays, additional antibodies were used as specified: CD107a-FITC (BD Biosciences, clone H4A3, 1:9, RRID: AB_396134), CD45 PerCP (BD Biosciences, clone 2D1, 1:20, RRID: AB_2868830), CD3-VioBlue (Miltenyi Biotec, 1:20, RRID: AB_2725961), CD8 APC-Cy7 (BD Biosciences, clone SK1, 1:50, RRID: AB_396892), and IFNγ-APC (BD Biosciences, clone B27, 1:100, RRID: AB_398580). For staining, cells were washed and resuspended in fluorescence-activated cell sorting (FACS) stain solution [Hank’s Balanced Salt Solution (HBSS), 20% (vol/vol) FBS, 0.1% (wt/vol) NaN_3_ (CAS 26628-22-8)], incubated with antibodies for 30 min at 4 °C, followed by secondary stain if necessary, and then washed and run on the MACSQuant (Miltenyi Biotec, RRID: SCR_020268). Flow data were analyzed with FCS Express 4 (De Novo Software, RRID: SCR_016431).

### Protein Expression and Purification.

Human IL13 and the IL13 variants were cloned into the pAcGP67-A vector (BD Biosciences) in frame with an N-terminal gp67 signal sequence and a C-terminal hexahistidine tag and produced using the baculovirus expression system, as described in LaPorte et al. (34). Baculovirus stocks were prepared by transfection and amplification in *Spodoptera frugiperda* (Sf9) cells grown in SF900II media (Invitrogen), and protein expression was carried out in suspension *Trichoplusiani* (High Five) cells grown in InsectXpress media (Lonza). Following expression, proteins were captured from High Five supernatants after 48 h by nickel-nitrilotriacetic acid (nickel-NTA) agarose (Qiagen) affinity chromatography, concentrated, and purified by size exclusion chromatography on a Superdex 200 column (GE Healthcare), equilibrated in 10 mM HEPES (pH 7.2) containing 150 mM NaCl (CAS 7647-14-5). Recombinant cytokines were purified to greater than 98% homogeneity. For biotinylated receptor expression, IL13Rα1/IL13Rα2 ectodomains were cloned into the pAcGP67-A vector with C-terminal biotin acceptor peptide LNDIFEAQKIEWHW followed by a hexahistidine tag. Receptors were coexpressed with BirA ligase in the presence of excess biotin (10 µM). Protein concentrations were quantified by ultraviolet (UV) spectroscopy at 280 nm using a Nanodrop2000 spectrometer (Thermo Fisher Scientific, RRID: SCR_018042).

### Yeast Display of IL13.

General yeast display methodologies were modified from previously described protocols (48). Human IL13 complementary DNA (cDNA) was cloned into the yeast display vector pCT302 (Addgene, RRID: Addgene_41845). *Saccharomyces cerevisiae* strain EBY100 (MYA-4941, ATCC) was transformed with the pCT302_IL13 vector and grown for 2 days at 30 °C on synthetic defined medium with casamino acids (SDCAA) plates. Individual colonies of IL13-displaying yeast were grown overnight at 30 °C in SDCAA liquid media (pH 4.5), followed by induction in Sabouraud galactose casamino acid (SGCAA) media (pH 4.5) for 2 days at 20 °C. Yeast were stained with biotinylated IL13Rα1 or IL13Rα2, followed by incubation with streptavidin (R&D Systems, catalog BAF614, RRID: AB_2124420, and catalog BAF152, RRID: AB_356026) coupled to Alexa 647 dye (catalog S32357, Invitrogen). Fluorescence was analyzed on an Accuri C6 flow cytometer (RRID: SCR_019591).

### SPR.

SPR experiments were conducted on a Biacore T100 instrument using a Biacore streptavidin (SA) sensor chip (GE Healthcare, RRID: SCR_019679). Biotinylated IL13Rα1/IL13Rα2 was captured at a low density (50 to 100 response units [RUs]), and kinetics measurements were conducted at 30 µL/min. An unrelated biotinylated protein was immobilized as a reference surface for the SA sensor chip with matching RUs to the experimental surface. All measurements were made using threefold serial dilutions of IL13 agonists in 1x HEPES-buffered saline with surfactant P20 (HBS-P) with 0.1% bovine serum albumin (BSA)). The IL13Rα1/IL13Rα2 bound to the chip surface was regenerated with 7 mM glycine (pH 3.0) (CAS 56-40-6) and 250 mM NaCl. Kinetic parameters were determined using 120 to 190 s of IL13 agonist association time and 20 to 1,200 s of dissociation time. All data fitting was performed using Biacore T100 evaluation software version 2.0 with a 1:1 Langmuir binding model.

### CAR Construct.

The codon-optimized IL13 (E12Y)–variant CAR sequence was previously described (26). The ribosomal skip *T2A* sequence (49) was fused by PCR splice overlap extension to the *CD19t* sequence obtained from the leader peptide to the transmembrane-spanning components (i.e., base pairs 1 to 972) of a *CD19*-containing plasmid. The *IL13*-variant and *T2A-CD19t* fragments were ligated into the previously described epHIV7 lentiviral vector (50). The *CD28* costimulatory sequence was inserted by splice overlap PCR, and then that construct underwent sequential site-directed mutagenesis using the QuikChange II XL kit (catalog 200521, Agilent Technologies) to generate the CAR variants.

### Isolation of Enriched Tn/mem Cells.

Blood products were obtained from healthy donors under protocols approved by the COH Internal Review Board. Peripheral blood mononuclear cells (PBMCs) were isolated by density gradient centrifugation over Ficoll-Paque (catalog 17-1440-02, GE Healthcare). PBMCs were incubated with clinical-grade anti-CD25 and anti-CD14 microbeads (catalog 200-070-211 and 200-070-121, Miltenyi Biotec) for 30 min at room temperature (RT) in X-Vivo15 media (catalog BW04-744Q, BioWhittaker) containing 10% FBS. CD25+ and CD14+ cells were then immediately depleted using the CliniMACS depletion mode according to the manufacturer’s instructions (Miltenyi Biotec, RRID: SCR_008984). After centrifugation, the unlabeled negative fraction of cells was resuspended in CliniMACS PBS/ethylenediaminetetraacetic acid (EDTA) buffer (catalog 200-070-026, Miltenyi Biotec) containing 0.5% human serum albumin (HSA) (catalog NDC 0944-0493-01, CSL Behring) and then labeled with clinical grade biotinylated-DREG56 monoclonal antibody (COH Center for Biomedicine and Genetics) at 0.1 μg/10^6^ cells for 30 min at RT. The cells were then washed and resuspended in a final volume of 100 mL CliniMACS PBS/EDTA containing 0.5% HSA. After 30 min of incubation with 1.25 mL of antibiotin microbeads (catalog 200-070-200, Miltenyi Biotec), the CD62L+ fraction (Tn/mem) was purified with positive selection on CliniMACS according to the manufacturer’s instructions (catalog 200-070-200, Miltenyi Biotec) and resuspended in X-Vivo15 media containing 10% FBS.

### Activation, Lentiviral Transduction, and Ex Vivo Expansion of CAR T Cells.

Tn/mem cells were stimulated with Dynabeads Human T-Expander CD3/CD28 (catalog 11141D, Invitrogen) at a 1:3 cell-to-bead ratio and transduced with lentivirus at a multiplicity of infection of 1.5 to 3 in X-Vivo15 containing 10% FBS and 100 μg/mL protamine sulfate (catalog NDC 63323-0229-15, APP Pharmaceuticals), 50 U/mL rhIL2 (catalog NDC 76310-0022-01, Clinigen), and 0.5 ng/mL rhIL15 (catalog 1013-050, CellGenix). Cultures were then maintained at 37 °C and 5% CO_2_, with addition of X-Vivo15 media containing 10% FBS as required to keep cell density around 6 × 10^5^ cells/mL, with cytokine supplementation 3 times a week. On day 7 of culture, the CD3/CD28 Dynabeads were removed from cultures using a DynaMag 5 magnet (catalog 12303D, Invitrogen). T cell lines were enriched with EasySep CD19 selection kit II (catalog 17854, Stemcell) around day 14 and propagated for 19 to 24 days prior to cryopreservation.

### Cytokine Production Assays.

For degranulation and intracellular IFN-γ assessment, CAR T cells and tumors were cocultured at a 1:1 E:T ratio in X-Vivo15 containing 10% FBS without cytokines. CD107a (BD Biosciences, RRID: AB_396134) and Golgi Stop [BD Biosciences, 1:1,500 (vol/vol), RRID: AB_2869009] were added to the coculture prior to the 5-h incubation at 37 °C. Subsequently, the intact cells were stained with human CD45, CD3, CD8, CD19, and IL13Rα2 antibodies. The cells were then fixed, permeabilized using Cytofix/Cytoperm (BD Biosciences, RRID: AB_2869008) per manufacturer’s instructions, stained for IFN-γ, and analyzed. For quantification of IFN-γ and IL2 from functional killing assays, the supernatants from 24-h cocultures (1:1 E:T ratio) were collected and measured using a Luminex assay (catalog LHC0001M, Thermo Fisher Scientific).

For enzyme-linked immunosorbent assays (ELISAs), T cells were cultured overnight at 5 × 10^3^ effectors per well in flat-bottom, 96-well plates (catalog 3361, Corning) that had been coated with 500, 250, 125, 62.5, or 31.25 ng/well rhIL13Rα1-Fc chimera (catalog 146-IR-100, R&D Systems) or IL13Rα2-Fc chimera (catalog 7147-IR-100, R&D Systems). Supernatants were then evaluated for IFN-γ levels using the Legend Max ELISA kit (BioLegend, RRID: AB_10896943) per manufacturer’s instructions.

### Cytotoxicity Assays.

T cells and tumors were cocultured at 1:10 and 1:4 E:T ratios in X-Vivo15 containing 10% FBS without the addition of cytokines in flat-bottom, 96-well plates (catalog 07-200-90, Corning) for 2 days. For extended killing assays, effectors and targets were cocultured at a 1:50 ratio for 7 days in the absence of cytokines, with fresh media replenishment every 3 to 4 days. At the end of the assay, adherent tumors were harvested enzymatically using trypsin (catalog MT25051CI, Corning). Cells were then stained with human CD45, CD8, CD19, and IL13Rα2 and assessed by flow. Tumor killing by CAR T cells was calculated by comparing viable CD45-negative cell counts relative to that observed with mock (nontransduced) T cells.

### Xenograft Models.

All mouse experiments were approved by the COH Institute Animal Care and Use Committee. In an orthotopic model, ffLuc^+^ PBT030-2 cells (1 × 10^5^) were stereotactically implanted into the right forebrain of NSG mice on day 0. Mice were then treated intratumorally with 0.3 × 10^6^ CAR T cells (0.3 × 10^6^ to 0.36 × 10^6^ total T cells, depending on the percentage that were CAR expressing) or 0.36 × 10^6^ mock transduced T cells (to match the highest number of total T cells injected), as indicated for each experiment. Groups of mice were then monitored for tumor engraftment by Xenogen (RRID: SCR_020901) noninvasive optical imaging as previously described (8) or for survival, with euthanasia applied according to the American Veterinary Medical Association Guidelines. An s.c. model was established by injecting HT1080 tumors (5 × 10^5^, 50 µl) in 50% (vol/vol) Matrigel (catalog 354234, Corning) to the flank of NSG mice. To assess IL13Rα1-targeted antitumor activity, 4 days after s.c. HT1080 IL13Rα1-IL4Rα tumor engraftment, CAR T cells (5 × 10^6^) were injected intratumorally and tumor sizes were monitored by caliper. To perform the Winn assay, A549 tumors (1 × 10^5^) and CAR T cells (1 × 10^6^) or mock T cells were coincubated in culture media at 37 °C for 2 h. Cell mixtures were then mixed with 50% (vol/vol) Matrigel and injected into the flank of NSG mice. To assess T cell trafficking, 8 days after s.c. HT1080 IL13Rα1-IL4Rα and HT1080 IL13Rα2 tumor engraftment, ffLuc-engineered CAR T cells (10 × 10^6^) were adoptively administered i.v., and CAR T cell distribution was monitored by optical imaging. A tumor dissociation kit was used to process tissue for flow cytometry (Miltenyi Biotec, RRID: SCR_020285). To interrogate the IT persistence of CAR T cell variants, M202 tumor cells were engrafted s.c. into the flank of NSG mice. Mice groups were sorted based on size. Upon tumor engraftment, mice were sorted based on tumor size, and 2 × 10^6^ ffLuc-engineered CAR T cells were injected intratumorally. Persistence was monitored using IVIS^®^ bioluminescence imaging and subsequently with flow cytometric analysis on the dissociated tumors.

### Statistical Analysis.

Statistical significance was determined by Student’s *t* test (two groups), two-way ANOVA with multiple comparisons, one-way ANOVA (more than three groups, Bonferroni adjustment), or log rank (Kaplan-Meir survival curve, Mantel-Cox adjustment) in GraphPad Prism (GraphPad Software, RRID: SCR_002798). Significance levels are represented as *P < 0.05, **P < 0.01, and ***P < 0.001; N.S. indicates not significant.

## Supplementary Material

Supplementary File

## Data Availability

All study data are included in the article and/or *SI Appendix*.
