# Supplementary material for: Engineered IL13 variants direct specificity of IL13Rα2-targeted CAR T cell therapy
Source: Proc Natl Acad Sci U S A. 2022 Aug 8;119(33):e2112006119. doi: 10.1073/pnas.2112006119 (PMC9388138; doi:10.1073/pnas.2112006119)
Supplement: Supplementary File [file pnas.2112006119.sapp.pdf]

Supplemental Figure 1

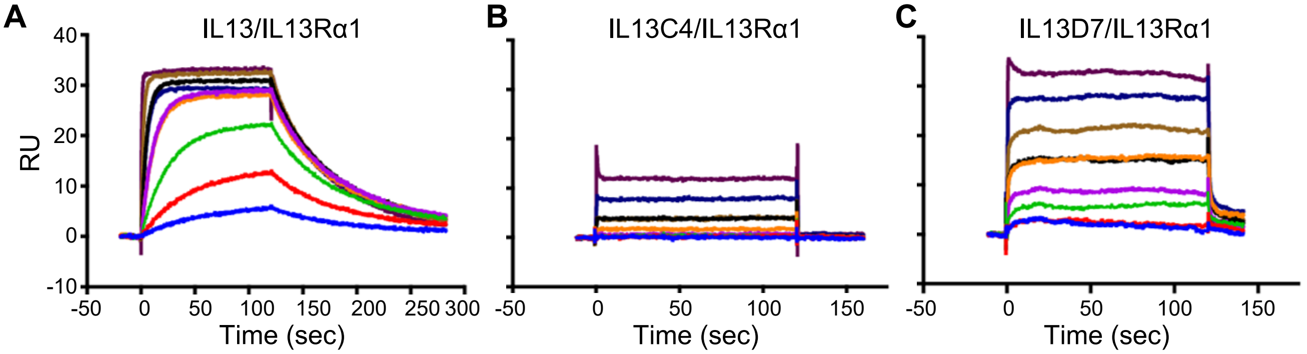

K_{on} [$M^{-1}sec^{-1}$]	K_{off} [sec^{-1}]	K_D [nM]
5.21×10^6	2.2×10^{-2}	4.38

K_{on} [$M^{-1}sec^{-1}$]	K_{off} [sec^{-1}]	K_D [nM]
N.D.	N.D.	36,000

K_{on} [$M^{-1}sec^{-1}$]	K_{off} [sec^{-1}]	K_D [nM]
N.D.	N.D.	4,100

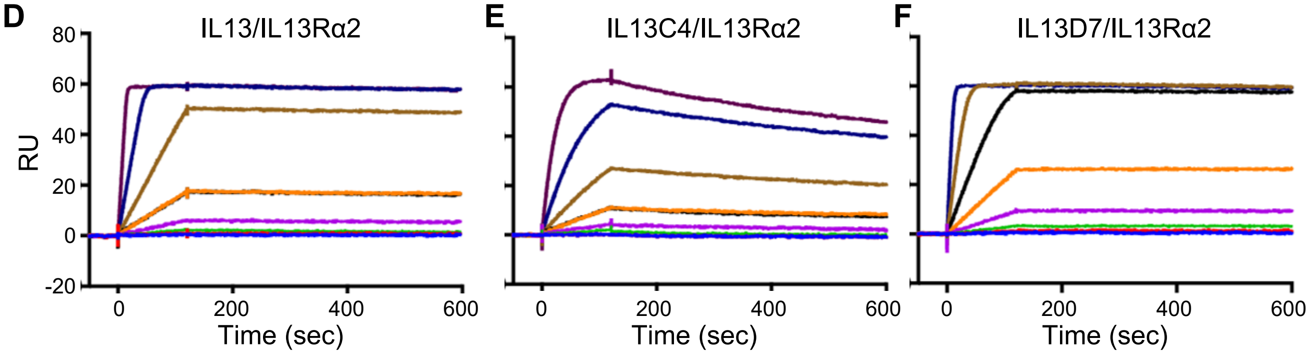

K_{on} [$M^{-1}sec^{-1}$]	K_{off} [sec^{-1}]	K_D [nM]
5.00×10^7	8.4×10^{-5}	0.001

K_{on} [$M^{-1}sec^{-1}$]	K_{off} [sec^{-1}]	K_D [nM]
1.94×10^6	7.64×10^{-6}	0.393

K_{on} [$M^{-1}sec^{-1}$]	K_{off} [sec^{-1}]	K_D [nM]
1.77×10^7	5.37×10^{-5}	0.003

Supplemental Figure 1. IL13Rα1 and IL13Rα2 binding affinities of the IL13 variants. Ratio of surface plasmon resonance measurements of IL13, IL13 C4, and IL13 D7 compared to (A-C) IL13Rα1 and (D-F) IL13Rα2. IL-13Rα1 and IL13Rα2 were immobilized on the surface of the chip and the indicated doses of IL13 or the two IL13 mutants were flowed.

Supplemental Figure 2

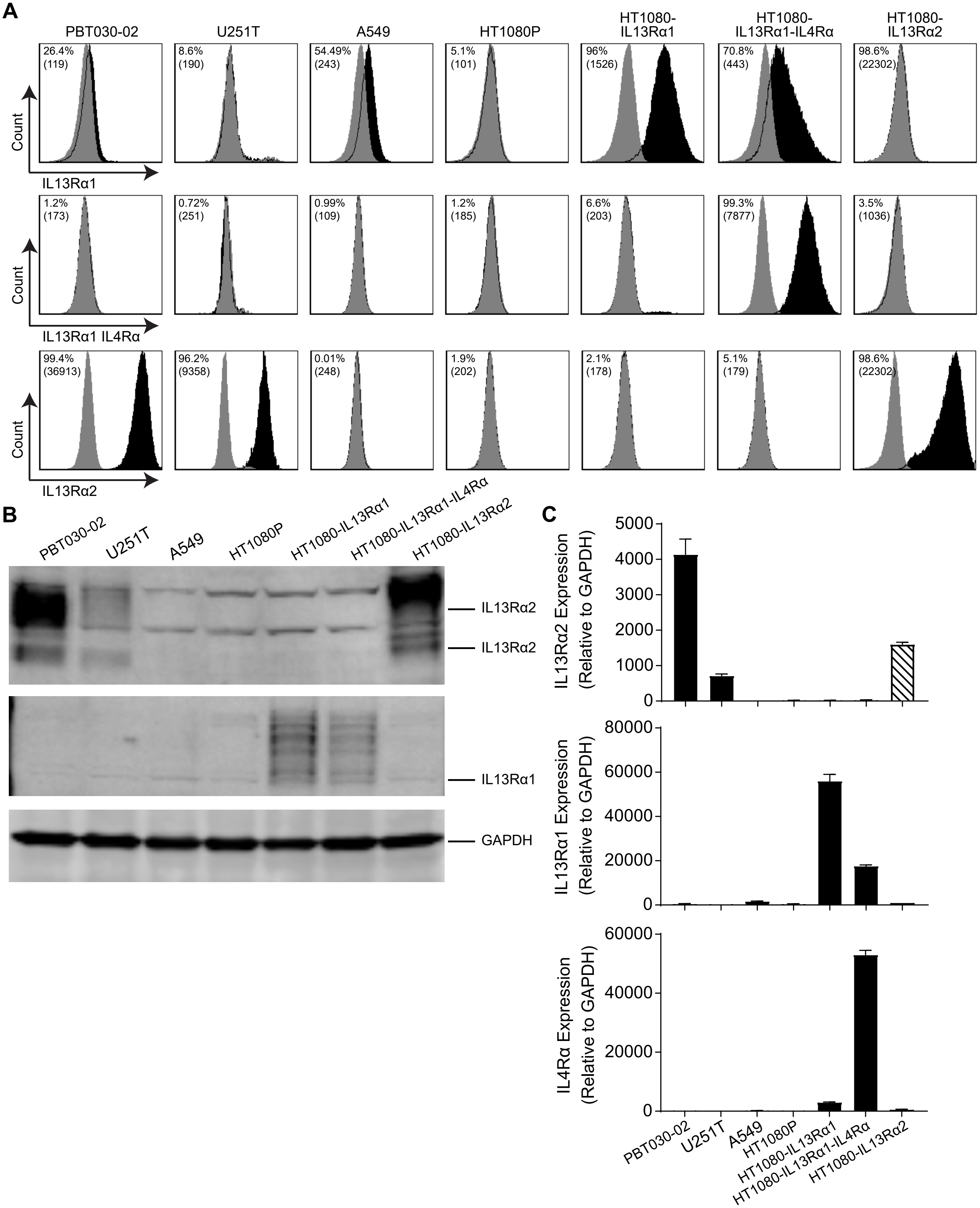

Supplemental Figure 2.
Detection of IL-13Rα2, IL-13Rα1, and IL-4R. **A,** Flow cytometry histograms showing either IL-13Rα2, IL-13Rα1, or IL-4R expression in the following adherent human cancer cell lines: PBT030-02, U251T, A549, HT1080P, HT1080-IL13Rα1, HT1080-IL13Rα1/IL4Rα, and HT1080-IL13Rα2. The cancer cell lines were enzymatically dissociated into single cells and immediately stained with antigen markers (IL13Rα2, IL13Rα1, IL4Rα) at 4C° in the dark for 30 min. Cells were then assessed by flow cytometry and the antigen expression of viable tumors were displayed as histograms. Values located within each histogram represent the % expression with the mean fluorescence intensity (MFI) in parentheses. **B,** Western blot showing the protein expression of IL13Rα2 and IL13Rα1, with β-actin as a loading control. **C,** qPCR analysis showing the mRNA expression of *IL13Rα2*, *IL13Rα1*, *IL4Rα* and codon optimized *IL13Rα2*. For both Western blot and qPCR, protein or mRNA lysates were isolated, respectively, from the previously mentioned cancer cell lines. All data shown are means ± sem.

Supplemental Figure 3

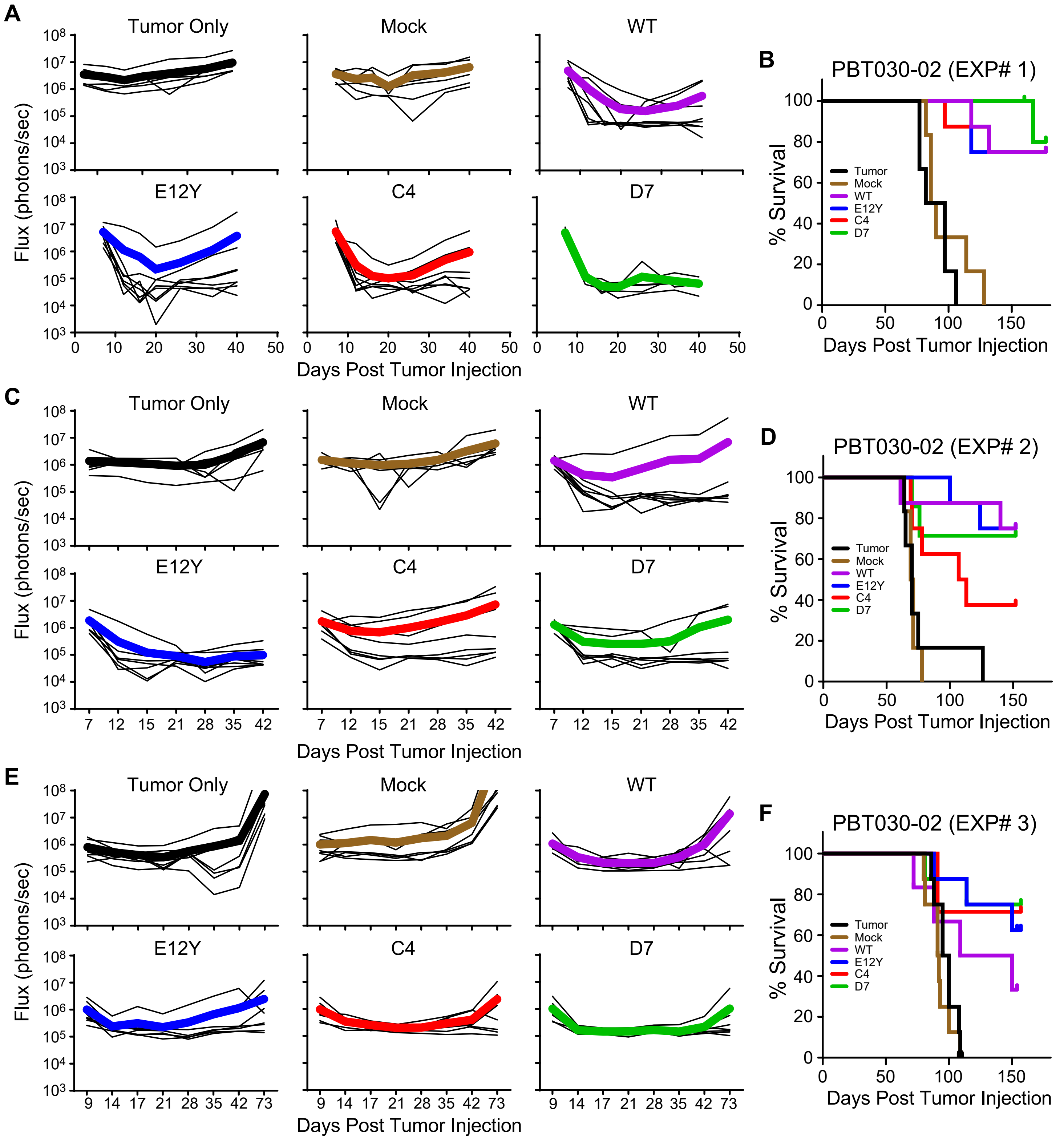

Supplemental Figure 3. IL13-E12Y, -C4 and -D7 mutein CARs exhibit similar levels of therapeutic efficacy *in vivo* against a human glioma model. (A, C, E) Quantification of ffLuc+ flux (photons/sec) show C4 and D7 IL13 mutein CAR T cells have comparable potency to the WT and E12Y single mutant IL13-CARs. Solid lines represent the flux values of individual mice while the colored solid lines represent the average flux of that treatment cohort. Data are representative of three independent experiments (n= 6-8). **(B, D, F)** Kaplan-Meier survival curves for the corresponding adjacent flux values of the individual demonstrating comparable survival and tumor relapse rate for mice treated with the IL13-CAR variants, with IL13-WT, -E12Y, -C4, and -D7 CAR T cells all improving survival as compared to mock T cell treated and untreated mice ($p \leq 0.0001$ Mantle-Cox log rank test (n=6-8).

Supplemental Figure 4

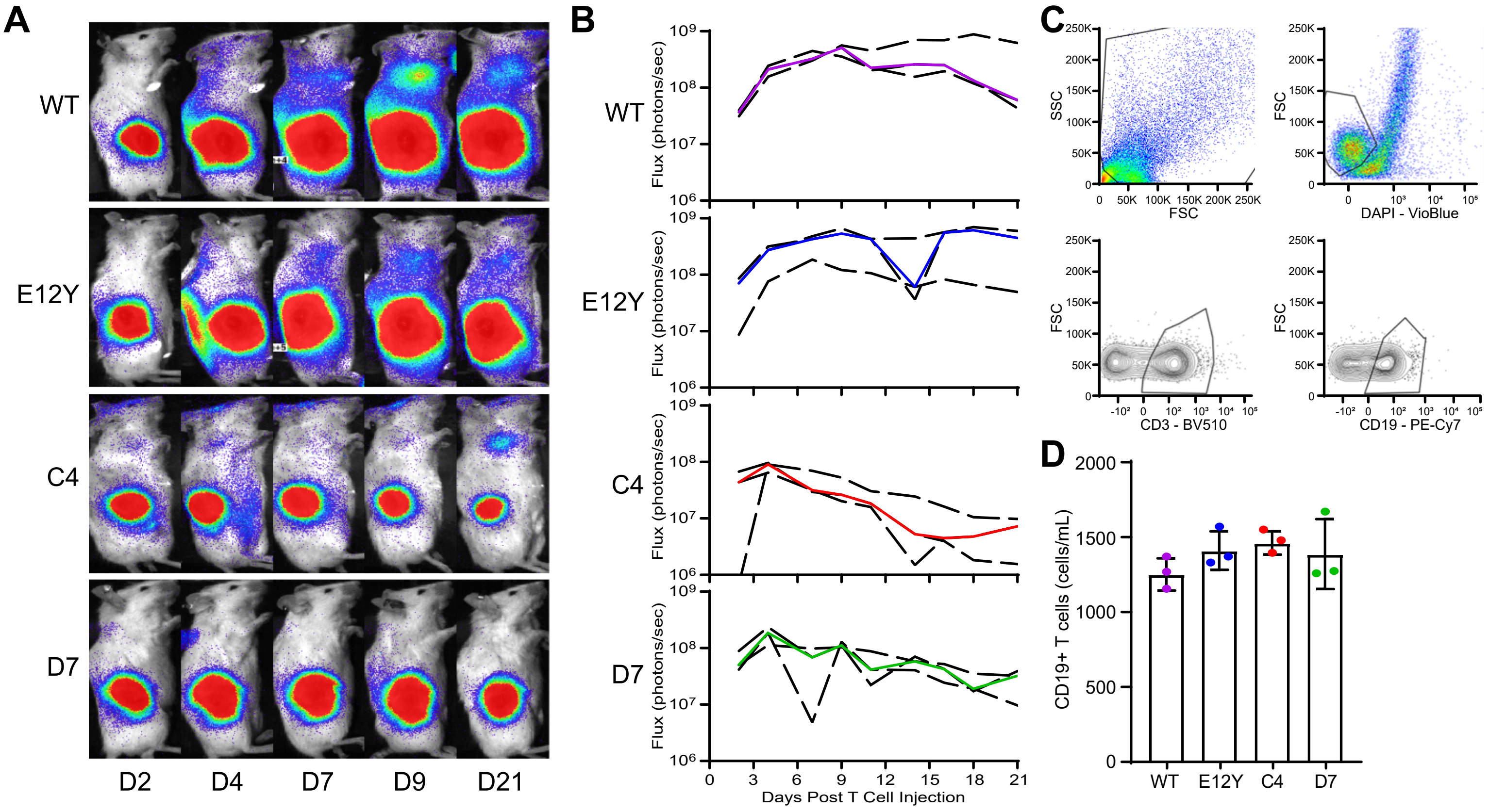

Supplemental Figure 4. IL13-C4 and -D7 mutein CARs exhibit reduced tumor burden *in vivo* against a human glioma model. (A) On day -33, 3 groups of mice (n=4) were injected with either 1 × 10^6, 2 × 10^6, or 5 × 10^6 M202 tumor cells. On day 0, the mice were resorted into 4 groups (n=3) and injected with 2 × 10^6 WT, E12Y, C4, or D7 variant CAR T cells that co-expressed ffluc. Luminescence imaging was carried out to track CAR T cell persistence. One representative mouse from each group is shown. **(B)** Quantification of ffluc+ flux (photons/sec) showing CAR T cell persistence. Dashed lines represent the flux values of individual mice while the colored solid lines represent the average flux for that treatment cohort. **(C)** At the experimental endpoint, tumors were excised and dissociated for flow cytometry analysis. The gating strategy for quantifying live CAR T cells (CD3+ CD19+) is shown. **(D)** Counts of CAR T cells in dissociated tumors were quantified by flow cytometry (mean ± SD; n=3).

Supplemental Figure 5

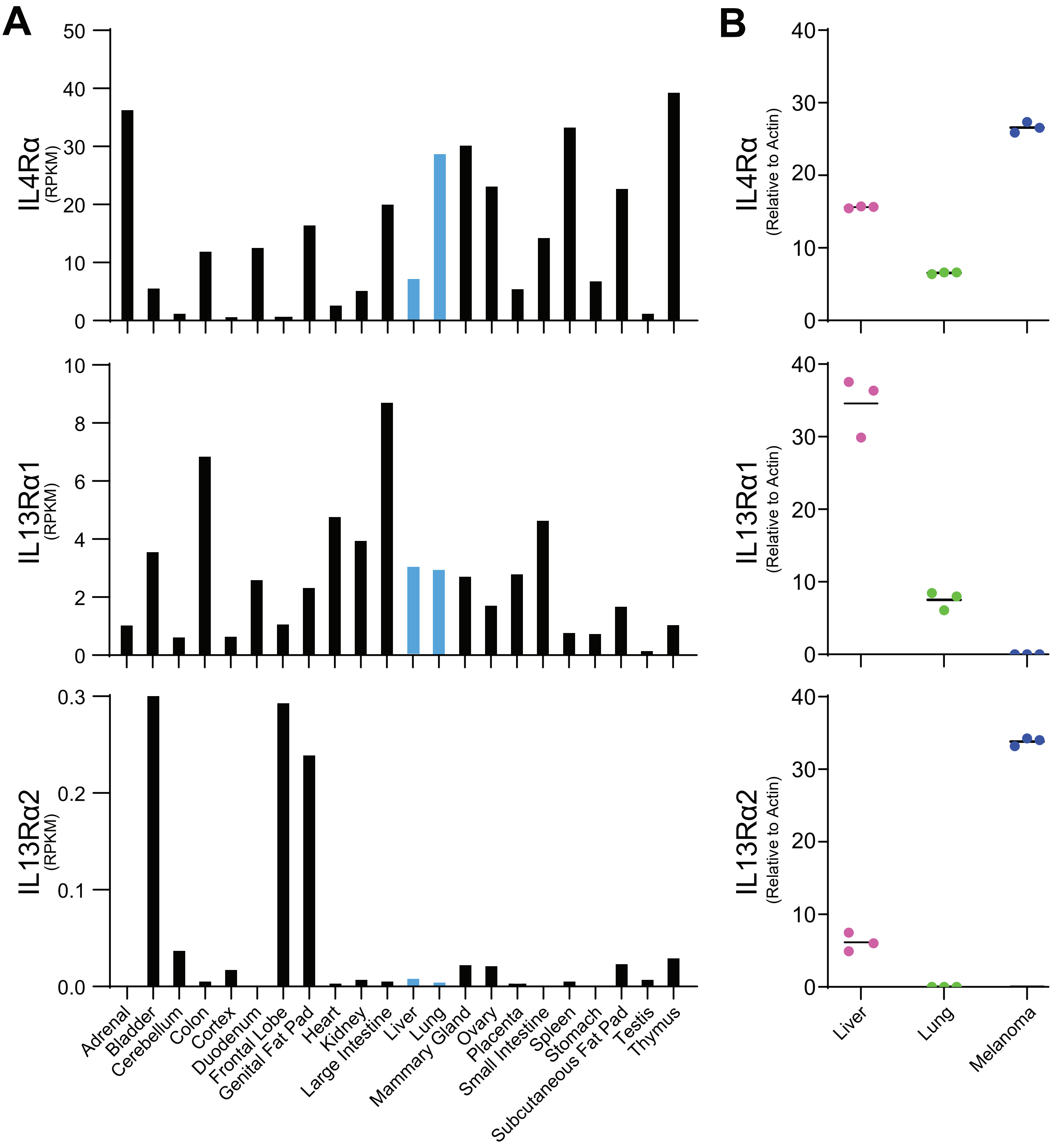

Supplemental Figure 5. Expression of IL4Rα, IL13Rα1, or IL13Rα2 in mouse tissue. (A) RNAseq data from the Encyclopedia of DNA Elements (ENCODE) shows expression levels of IL4Rα, IL13Rα1, or IL13Rα2 in C57BL/6 mouse tissues. The Y axis was scaled according to RPKM expression levels. **(B)** Quantitative PCR detected expression levels of IL4Rα, IL13Rα1, or IL13Rα2 in isolated liver and lung from NSG mice and a melanoma cell line (n=3 per group).

Supplemental Figure 6

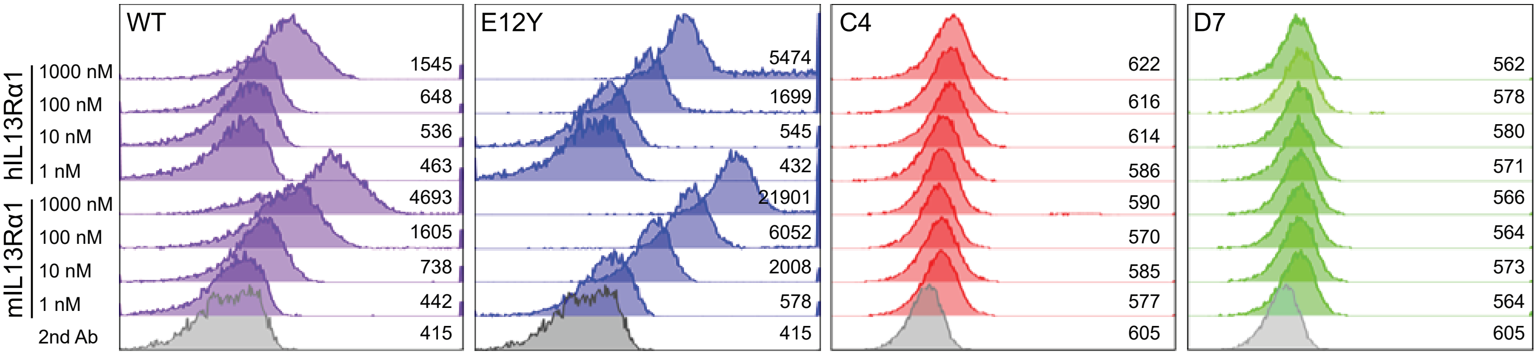

Supplemental Figure 6. Binding characteristics of human and murine IL13Rα1 with WT, E12Y, C4, and D7 muteins. Recombinant human and mouse IL13Rα1 were incubated with WT, E12Y, C4, or D7 CAR T cells at the concentrations shown. Binding was quantified by flow cytometry with MFI shown for each histogram. One representative trial for each variant is shown.

Supplemental Figure 7

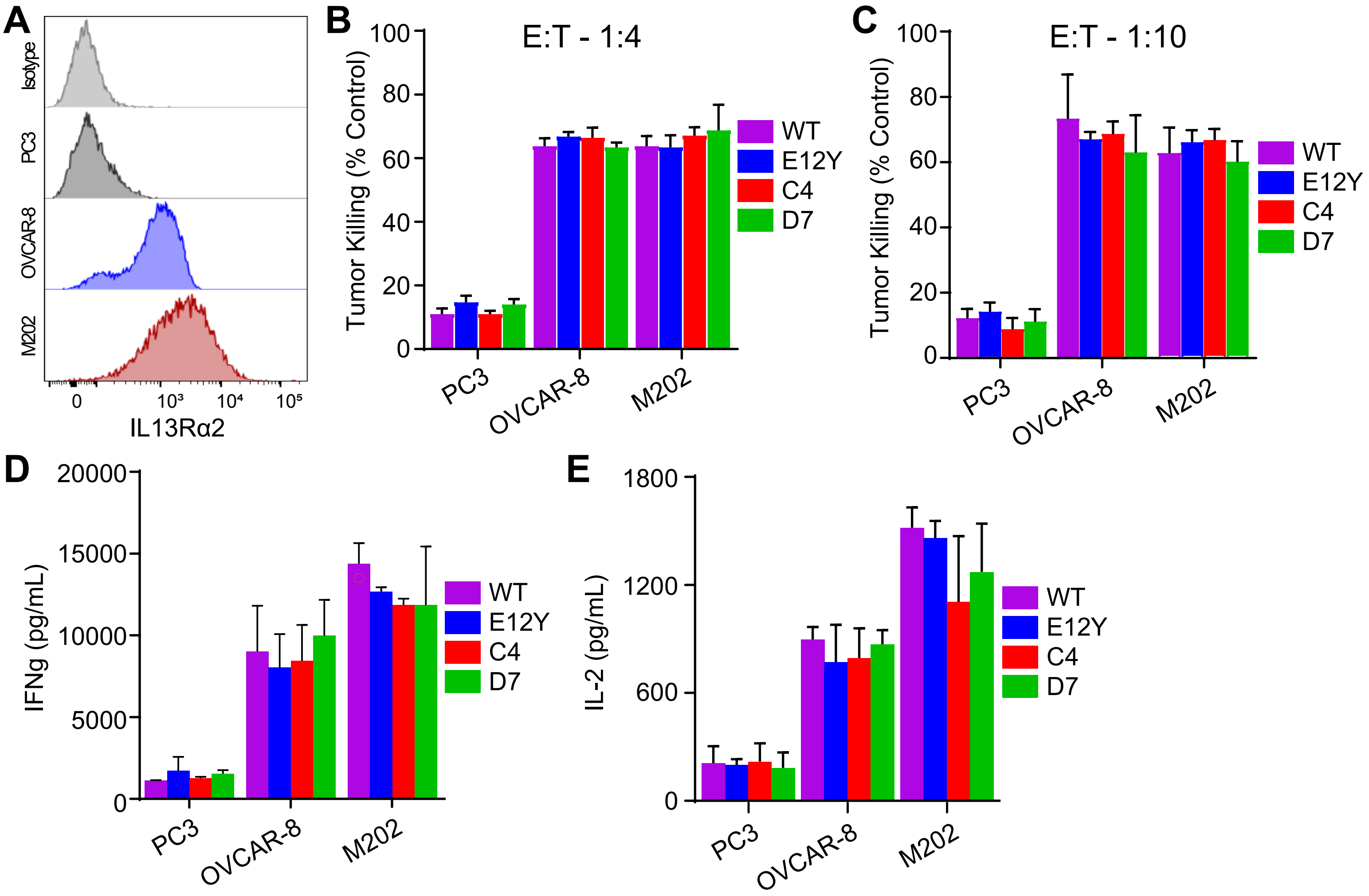

Supplemental Figure 7. Tumor killing and cytotoxicity of WT, E12Y, C4, or D7 muteins in other cancers. Muteins were co-cultured at a 1:4 or 1:10 effector to target (E:T) ratio with PC3 cells (do not express IL13Rα2) or IL13Rα2+ OVCAR-8 and M202 (n=4). After 24hrs, IFN-γ and IL-2 levels were measured by Luminex assay. **(A)** Expression of IL13Rα2 in PC3, OVCAR-8, and M202 tumor lines. **(B, C)** Tumor killing of PC3, OVCAR-8, and M202 tumor lines at an E:T of 1:4 (B) and 1:10 (C). **(D, E)** IFN-γ and IL-2 secretion by CAR T cells co-cultured with PC3, OVCAR-8, and M202 cell lines.

Supplemental Table 1

Organ	GEO Number	Data Coordination Center
Adrenal	GSM900188	ENCODE DCC
Bladder	GSM10000565	ENCODE DCC
Cerebellum	GSM10000576	ENCODE DCC
Colon	GSM900198	ENCODE DCC
Cortex	GSM10000563	ENCODE DCC
Duodenum	GSM900187	ENCODE DCC
Frontal Lobe	GSM10000562	ENCODE DCC
Genital Fat Pad	GSM900190	ENCODE DCC
Heart	GSM900199	ENCODE DCC
Kidney	GSM900194	ENCODE DCC
Large Intestine	GSM900189	ENCODE DCC
Liver	GSM900195	ENCODE DCC
Lung	GSM900196	ENCODE DCC
Mammary Gland	GSM900184	ENCODE DCC
Ovary	GSM900183	ENCODE DCC
Placenta	GSM10000565	ENCODE DCC
Small Intestine	GSM900186	ENCODE DCC
Spleen	GSM900197	ENCODE DCC
Stomach	GSM900185	ENCODE DCC
Subcutaneous Fat Pad	GSM900191	ENCODE DCC
Testes	GSM900193	ENCODE DCC
Thymus	GSM900192	ENCODE DCC

Supplemental Table 1. ENCODE data sets. Organs, GEO numbers, and data coordination centers were obtained from the ENCODE website (gene IDs: IL13Ra2 – 16165, IL13Ra1 – 16164, and IL4Ra – 16190).
